# Potential and Limits of Kidney Cells for Evaluation of Renal Excretion

**DOI:** 10.3390/ph14090908

**Published:** 2021-09-07

**Authors:** Christian Lechner, Ursula Mönning, Andreas Reichel, Gert Fricker

**Affiliations:** 1 Institute of Pharmacy and Molecular Biotechnology, Ruprecht-Karls-University, 69120 Heidelberg, Germany; Christian.Lechner@nuvisan.com; 2 Bayer Pharma AG, Research Pharmacokinetics, 13353 Berlin, Germany; ursula.moenning@bayer.com (U.M.); andreas.reichel@bayer.com (A.R.)

**Keywords:** renal excretion, kidney cell lines, marker enzymes, transport proteins

## Abstract

A large number of therapeutic drugs, herbal components and their metabolites are excreted by the kidneys. Therefore, generally applied models for estimating renal excretion, including freshly isolated rat proximal tubule cells, cultured tubule cells and immortalized kidney cell lines MDCKII, NRK-52E, IHKE-1 and Caki-1, were investigated regarding their predictive potential for active renal transport. Cultured proximal tubule cells showed an epithelial cell-like morphology and formed tight monolayers. However, mRNA expression analyses and immunohistochemical studies revealed patterns of tight junction proteins that were notably different from freshly isolated cells and distinct from those in vivo. High levels of mannitol permeation were found in NRK-52E, IHKE-1 and Caki-1 cells, suggesting that they are not suitable for bidirectional transport studies. Cultured cells and freshly isolated cells also differed in proximal tubule markers and transport proteins, indicating that cultured primary cells were in a state of dedifferentiation. Cell lines MDCKII, NRK-52E, IHKE-1 and Caki-1 did not accurately reflect the characteristics of proximal tubules. The expression patterns of marker and transport proteins differed from freshly isolated primary cells. In summary, each of these models has profound disadvantages to consider when adopting them reliable models for the in vivo situation. Thus, they should not be used alone but only in combination.

## 1. Introduction

In general, pharmacological activity occurs when the active agents or the active metabolites reach and sustain sufficiently high levels at their site of action. Since a large percentage of therapeutic drugs, herbal components and their metabolites is excreted by the kidneys, a full understanding of the excretion process is necessary for better evaluating the target site concentration and therapeutic response. Thereby, besides glomerular filtration, active secretion from peritubular capillaries across proximal tubular cells into the tubular lumen is of great importance. Proximal tubular cells are characterized by the expression of specific marker genes encoding, e.g., villin 1 (VIL1), γ-glutamyl transferase 1, alanine aminopeptidase and alkaline phosphatase liver/bone/kidney [1,2,3]. The typical barrier function of the proximal tubule epithelium is determined by the formation of adherens junctions and zonulae occludens or tight junctions. These are multiprotein complexes, including occludin (OCLN) and members of the claudin (CLDN) family, mainly claudin-2, claudin-7 and claudin-10 [4,5,6]. An associated protein is tight junction protein 1 (TJP1), also known as ZO-1, connecting the tight junctions with the actin cytoskeleton of the cell [7].

**Figure 1:**
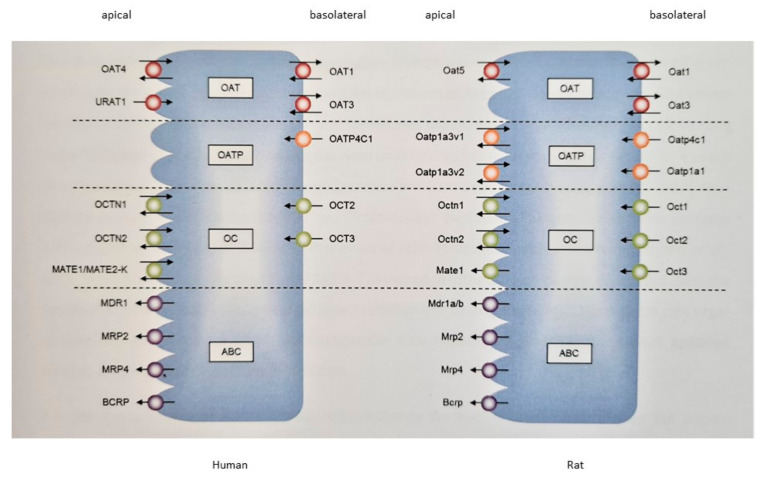
Proteins relevant for renal drug transport in humans and rats. OAT/Oat: Organic anion transporter; URAT: Urate transporter; OATP/Oatp: Organic anion transporting polypeptide; OC: Organic cation; OCTN/Octn: Organic cation transporter, novel; MATE/Mate: Multidrug and toxin extrusion protein; ABC: ATP-binding cassette; MDR/Mdr: Multidrug resistance; MRP/Mrp: Multidrug resistance-related protein; BCRP/Bcrp: Breast cancer resistance protein.

In addition, proximal tubule cells express a variety of transport systems mediating the active uptake and excretion of organic cations and anions (Figure 1) across the basolateral and apical membranes. Uptake transporters belong to the superfamily of solute carrier transporters (SLC) mediating facilitated transport along an electrochemical gradient or a secondary active transport against a gradient. The efflux transporters are mainly members of the ABC transporter superfamily.

Important transporters for the basolateral uptake of organic cations include organic cation transporters (OCTs) belonging to the SLC22 subfamily. In humans, OCT2 (SLC22A2) plays an important role in the excretion of cationic drugs; in rats, Oct1 (Slc22a1) is also important. The basolateral uptake of organic anionic drugs is mediated by organic anion transporters (OATs), OAT1 (SLC22A6) and OAT3 (SLC22A8) from the SLC22 subfamily, and organic anion transporting polypeptide (OATP), OATP4C1 (SLCO4C1). OAT2 (SLC22A7), which is also expressed in the apical membrane, appears to be of minor importance for the uptake of anionic drugs [8]. In the proximal tubule cells of rats, Oatp1a1 (Slco1a1) is also involved in anion uptake [9,10]. 

Efflux across the apical membrane is primarily mediated by primary active ATP-binding cassette (ABC) transporters. In humans, p-glycoprotein (MDR1, ABCB1), breast cancer resistance protein (BCRP, ABCG2) and multidrug resistance-related proteins (MRPs) MRP2 (ABCC2) and MRP4 (ABCC4) are of particular importance. In addition, multidrug and toxin extrusion (MATE) transporters MATE1 (SLC47A1) and MATE2-K (SLC47A2) of the SLC47 subfamily play a role in the extrusion of cationic drugs [11]. Reabsorption in the tubular membrane is mediated by the organic cation transporter/novel (OCTN) transporters OCTN1 (SLC22A4) and OCTN2 (SLC22A5), urate transporter (URAT) URAT1 (SLC22A12), the peptide transporters (PEPT) PEPT1 (SLC15A1) and PEPT2 (SLC15A2) and human OAT4 (SLC22A9), as well as the rat-specific Oatp1a3 (Slco1a3).

For investigation of such transport processes, several cell-based in vitro models are available, which allow high throughput at relatively low costs [12]. In order to study the function of distinct proteins, very often, cells overexpressing a single, two or even more transporters are used. Popular host cells are Madin-Darby canine kidney (MDCK), human embryonic kidney 293 (HEK293) or Chinese hamster ovary (CHO) cells. A potential drawback of such overexpression systems is the presence of endogenous transporters with similar substrate specificity or alterations in the expression patterns after transfection [13,14,15]. In addition, many compounds are transported by different transporters in parallel, making an in vitro/in vivo comparison difficult. 

Therefore, the aim of the present work was an investigation of several renal cell-based in vitro systems with respect to their suitability to study renal drug transport (Table 1). The focus of this effort was on the comparison of different cell lines as well as primary rat proximal tubule cells (PRPTCs) with respect to monolayer formation, expression of marker proteins, tight junction proteins and transport proteins.

## 2. Results

### 2.1. Phenotypic Characterization, Cell Morphology and Monolayer Integrity

**Figure 2:**
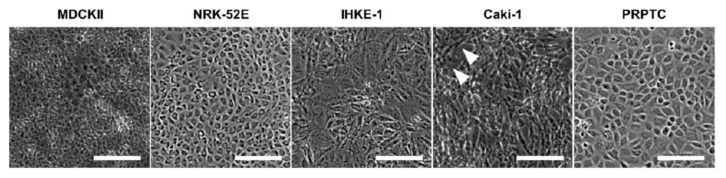
Phase contrast images of the renal cell lines MDCKII, NRK-52E, IHKE-1 and Caki-1, and of cultured PRPTCs. All cells were cultured on chambered glass slides for 5 days under standard conditions. Under these conditions, MDCKII, NRK-52E, IHKE-1 and PRPTC cells formed a confluent monolayer. In contrast, Caki-1 cells did not form a confluent monolayer and cell-free gaps (white triangles) remained even after extended culture periods. Scale bars = 200 µm.

Figure 2 shows the light microscopic characterization of the cultured cells. All cells formed monolayers within 5 days of culture. In particular, PRPTCs as well as NRK-52E-cells developed a cuboidal structure, whereas the human cell lines IHKE-1 and Caki-1 were rather irregular and more elongated. With the exception of Caki-1 cells, all cell lines as well as PRPTCs formed uniform monolayers, reaching confluency within 5 days in culture. Even at longer culture periods (up to 8 days), confluency was not reached by Caki-1 cells. In addition, Caki-1 cells did not show contact inhibition and started to form multilayers.

### 2.2. Expression Analyses of Proximal and Distal Tubule Markers

**Figure 3:**
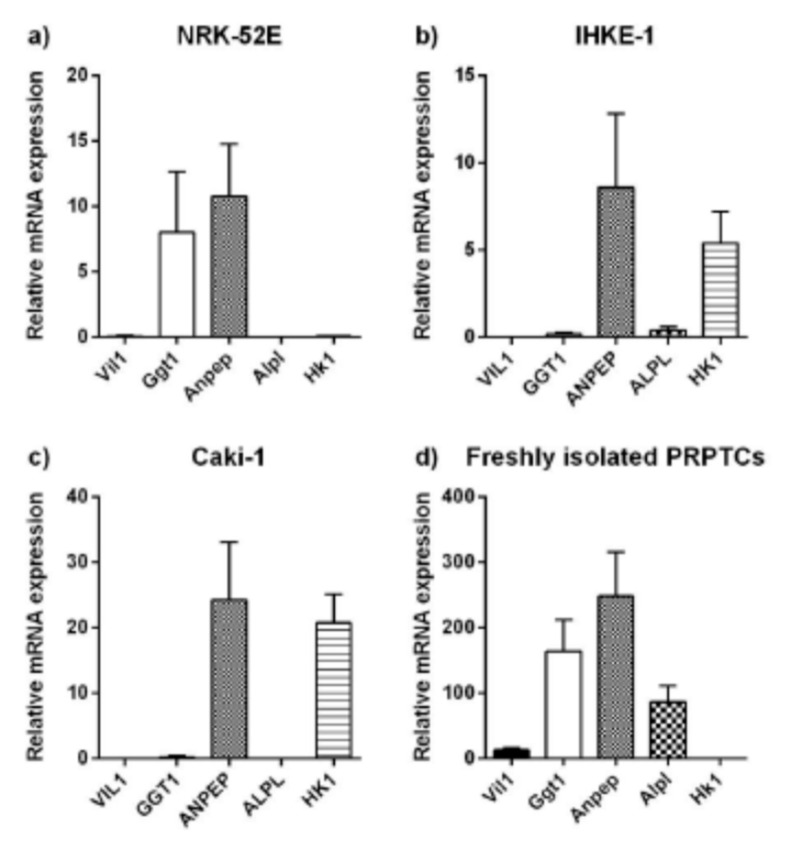
Relative mRNA expression of tubule marker proteins in the renal cell lines NRK-52E (**a**), IHKE-1 (**b**) and Caki-1 (**c**), and in freshly isolated PRPTCs (**d**). The mRNA expression of each gene was normalized to GAPDH/Gapdh expression. Each bar represents the mean ± SD (*n* = 3).

**Figure 4:**
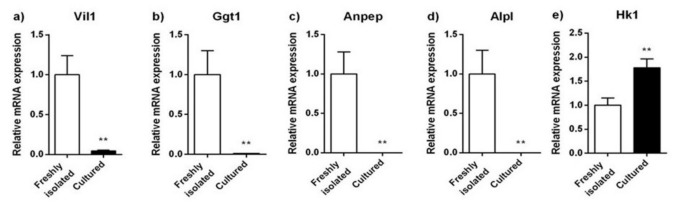
Relative mRNA expression of the tubule marker proteins Vil1 (**a**), Ggt1 (**b**), Anpep (**c**), Alpl (**d**) and Hk1 (**e**) in freshly isolated and cultured PRPTCs. The mRNA expression of each gene was normalized to Gapdh expression and is shown in reference to the expression level of each gene in freshly isolated PRPTCs, which was set to 1. Each bar represents the mean ± SD (*n* = 3), ** *p* < 0.01.

In order to clarify the identity of the isolated PRPTCs, mRNA expression of differentiation markers was determined by qPCR. Villin 1 (VIL1/Vil1), γ-glutamyl transferase 1 (GGT1/Ggt1), alanine aminopeptidase (ANPEP/Anpep) and alkaline phosphatase (ALPL/Alpl) were determined as markers for proximal tubules, and hexokinase 1 (HK1/Hk1) was determined as a marker for distal tubules. The mRNA expression analysis is summarized in Figure 3. Generally, considerable amounts of mRNA of all proximal markers could only be detected in freshly isolated cells, whereas NRK-52E, IHKE-1 and Caki-1 cells showed only very low expression levels for at least one of the markers. In all cell lines, the brush border membrane enzyme alanine aminopeptidase showed the highest expression levels (Caki-1 > NRK-52E > IHKE-1). However, compared to the cell lines, the expression level of alanine aminopeptidase was 10–29-fold higher in freshly isolated PRPTCs. This difference was even more pronounced for alkaline phosphatase and γ-glutamyl transferase 1, with ca. 86–3000-fold and 21–800-fold higher mRNA expression levels in freshly isolated PRPTCs, respectively. Considerable amounts of villin 1 mRNA were only detected in the PRPTCs. Additionally, the two human cell lines, IHKE-1 and Caki-1, had relatively high expression of the distal tubule enzyme hexokinase 1, suggesting potential redifferentiation to a distal cell type. Compared with this, hexokinase 1 expression was only very low in freshly isolated cells. In addition to freshly isolated PRPTCs, the expression of the tubule markers was also determined in PRPTCs kept in culture for 5 days (Figure 4). A drastic reduction in mRNA could be observed (e.g., alkaline phosphatase ca. 900-fold or villin 1 ca. 25-fold). In contrast, expression of the distal marker hexokinase 1 was 1.8-fold increased.

### 2.3. Expression Analyses of Tight Junction Proteins and Paracellular Transport

**Figure 5:**
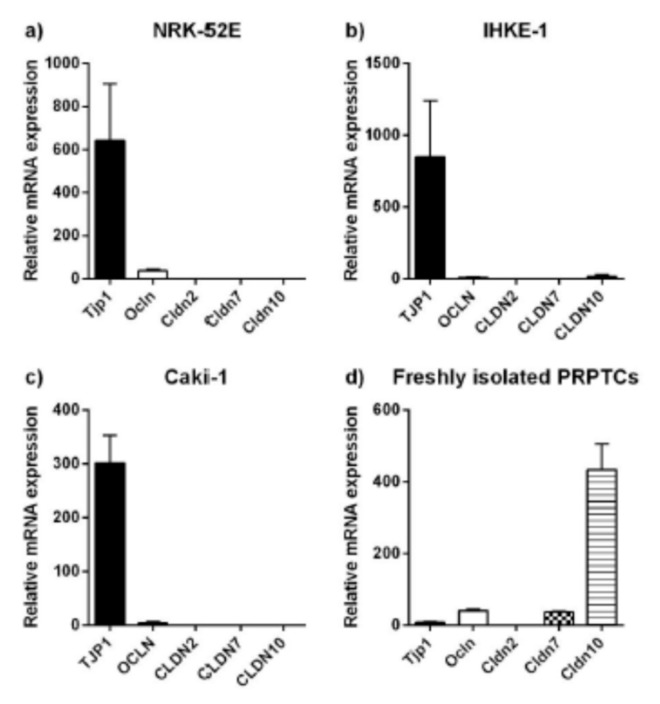
Relative mRNA expression of tight junction proteins in the renal cell lines NRK-52E (**a**), IHKE-1 (**b**) and Caki-1 (**c**), and in freshly isolated PRPTCs (**d**). The mRNA expression of each gene was normalized to GAPDH/Gapdh expression. Each bar represents the mean ± SD (*n* = 3).

The mRNA levels of tight junction proteins in the renal cell lines NRK-52E, IHKE-1 and Caki-1 as well as in freshly isolated PRPTCs are shown in Figure 5. Similar expression profiles were found in the three cell lines. The highest expression in NRK-52E, IHKE-1 and Caki-1 cells was detected for tight junction protein 1, while no or only very low levels of expression were found for claudin-2, -7 and -10. Freshly isolated PRPTCs showed a markedly different expression pattern. In these cells, claudin-10 had the highest expression and moderate levels of claudin-2 mRNA were detected as well. Moreover, the expression level of tight junction protein 1 was much lower than in the cell lines (ca. 35–100-fold).

**Figure 6:**
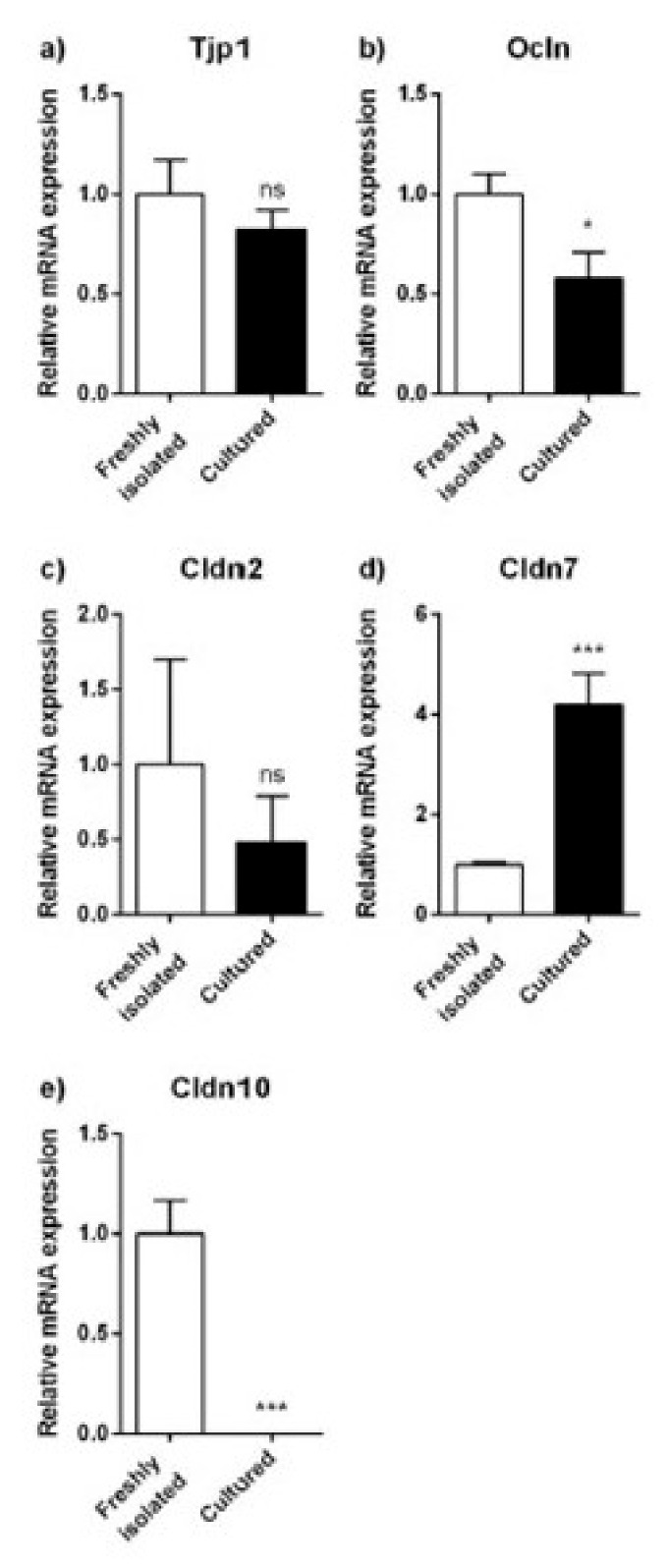
Relative mRNA expression of the tight junction proteins Tjp1 (**a**), Ocln (**b**), Cldn2 (**c**), Cldn7 (**d**) and Cldn10 (**e**) in freshly isolated and cultured PRPTCs. The mRNA expression of each gene was normalized to Gapdh expression and is shown in reference to the expression level of each gene in freshly isolated PRPTCs, which was set to 1. Each bar represents the mean ± SD (*n* = 3), * *p* < 0.05, *** *p* < 0.001, ns = not statistically different (*p* > 0.05).

The mRNA expression of the tight junction proteins was also studied in PRPTCs cultured for 5 days (Figure 6). Whereas the expression levels of tight junction protein 1, occludin and claudin-2 were not statistically different (*p* > 0.05) or only slightly decreased, a 4-fold increase in claudin-7 mRNA was observed. In contrast, the expression of claudin-10 was almost completely reduced in cultured PRPTCs (ca. 700-fold). 

**Figure 7:**
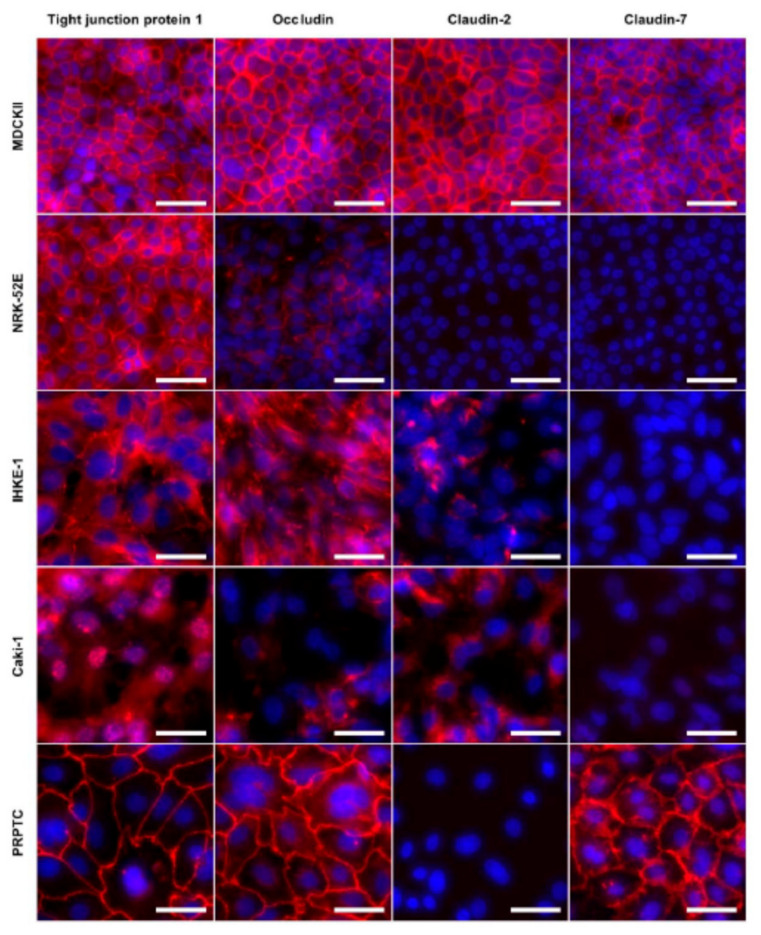
Expression of the tight junction proteins tight junction protein 1, occludin, claudin-2 and claudin-7 in the renal cell lines MDCKII, NRK-52E, IHKE-1 and Caki-1, and in cultured PRPTCs. All cells were cultured on chambered glass slides for 5 days under standard conditions. For immunofluorescent staining, cells were incubated with primary and Cy3-conjugated secondary antibodies (red) and DAPI (blue). Negative controls incubated only with secondary antibodies showed no staining (not shown). Scale bars = 100 µm.

In parallel to mRNA expression analysis, tight junction proteins were also visualized by immunofluorescent staining of the cell monolayers (Figure 7). A summary of the expression patterns of tight junction proteins in the cell lines and cultured PRPTCs is shown in Table 2. Distinct signals of tight junction protein 1, occludin, claudin-2 and claudin-7 at the cell–cell borders were detected throughout the monolayers of these cells, as shown in Figure 7. In comparison, the renal cell lines NRK-52E, IHKE-1 and Caki-1 showed diverging expression patterns. While tight junction protein 1 and occludin could be detected in all three cell lines, at least one of the claudins was absent. A claudin-2 signal could be detected in monolayers of IHKE-1 and Caki-1 cells but it was mainly found in the cytosol and not predominantly located at the cell–cell borders. In Caki-1 cells, this was also true for the expression of tight junction protein 1 and occludin, indicating non-functional tight junctions. In contrast to the cell lines NRK-52E, IHKE-1 and Caki-1, cultured PRPTCs and the MDCKII cell line did express claudin-7 in addition to tight junction protein 1 and occludin. The signal of all three detected tight junction proteins was strongest at the cell–cell borders. No signal was found for claudin-10 in any of the tested cells.

**Figure 8:**
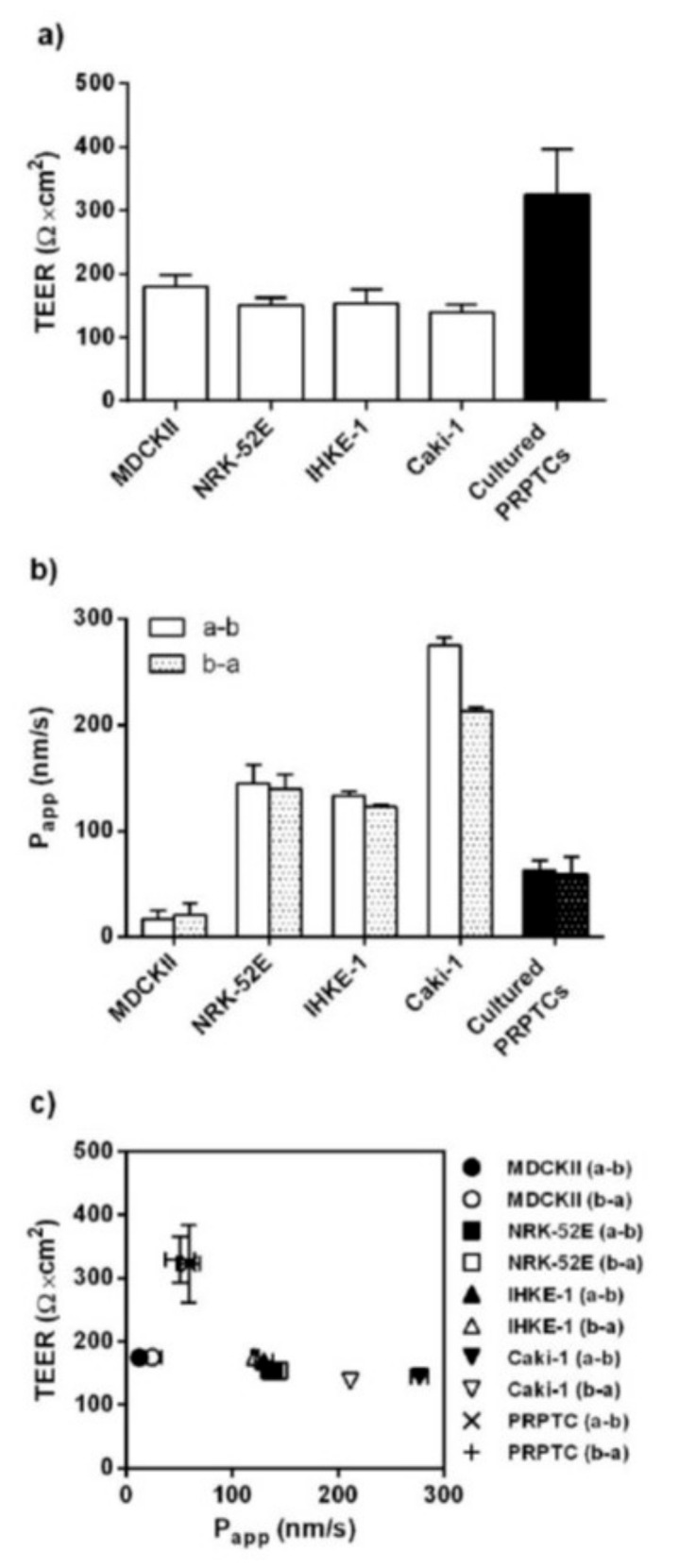
TEER values (**a**) and apparent permeability coefficients (P_app_) of [^14^C]-mannitol (**b**) in the renal cell lines MDCKII, NRK-52E, IHKE-1 and Caki-1 and in cultured PRPTCs. All cells were cultured on filter inserts for 5 days under standard conditions. For determination of P_app_ values, cell monolayers were incubated with 600 µM [^14^C]-mannitol (12.5 KBq/mL) for 120 min. Each bar represents the mean ± SD of 3 different sets of experiments. A correlation plot of TEER and P_app_ values is shown in (**c**).

In order to place the expression data of tight junction proteins into a functional context, the transepithelial electrical resistance (TEER) of the cell monolayers was determined (Figure 8a). The highest TEER value was measured with PRPTCs (326 Ω × cm^2^). TEER values of the cell lines MDCKII, NRK-52E, IHKE-1 and Caki-1 were slightly lower and ranged from 135 to 180 Ω × cm^2^. 

In addition to TEER measurements, the permeability of the cell monolayers was determined by measuring the bi-directional migration of [^14^C]-mannitol, a non-cell-permeable marker compound (Figure 8b). The lowest [^14^C]-mannitol flux was found in MDCKII cells, with P_app_ values of 16.5 nm/s and 20.7 nm/s for a–b and b–a transport, respectively. In comparison, [^14^C]-mannitol flux in NRK-52E and IHKE-1 cells was considerably higher, with P_app_ values ranging from 123 nm/s to 145 nm/s in both directions. With P_app_ values of 275 nm/s for a–b and 213 nm/s for b–a transport, Caki-1 cells showed the highest permeability for [^14^C]-mannitol. Moderate permeability was observed with monolayers of cultured PRPTCs; [^14^C]-mannitol flux was slightly higher than in MDCKII cells and lower than in the other cell lines. The efflux ratios were approximately 1 in all cells, indicating a passive transport process.

When the paracellular movement of [^14^C]-mannitol was plotted versus TEER values, no clear correlation could be seen (Figure 8c). In theory, low P_app_ values are associated with high TEER values, indicating tight monolayers (13,14). However, MDCKII cells showed the lowest P_app_ values, i.e., the lowest [^14^C]-mannitol flux, while, at the same time, TEER values were lower than in PRPTCs. Moreover, although varying P_app_ values were measured with NRK-52E, IHKE 1 and Caki-1 cells, the TEER values did not differ considerably and were similar in all three cell lines.

### 2.4. Expression of Transport Proteins

Several transport proteins play an essential role in the active uptake and excretion of drugs. They include the ABC transporters MDR1/Mdr1 (ABCB1/Abcb1), MRP2/Mrp2 (ABCC2/Abcc2), MRP4/Mrp4 (ABCC4/Abcc4) and BCRP/Bcrp (ABCG2/Abcg2), as well as uptake transport proteins belonging to the SLC22 and the SLCO families. In addition, the MATE transporters MATE1/Mate1 (SLC47A1/Slc47a1) and MATE2-K (SLC47A2) and PEPT1/Pept1 (SLC15A21/Slc15a1) and PEPT2/Pept2 (SLC15A2/Slc15a2) are relevant as well. Since these transporters show species differences, not all of them were studied in all cells. The expression of MATE2-K (SLC47A2) and OAT4 (SLC22A9) is limited to proximal tubules in humans and was therefore only studied in the cell lines IHKE-1 and Caki-1. The expression of Oat5 (Slc22a24), Oatp1a1 (Slco1a1) and Oatp1a3 (Slco1a3) is limited to rats and was only studied in the NRK-52E-cell line as well as in PRPTCs. 

**Figure 9:**
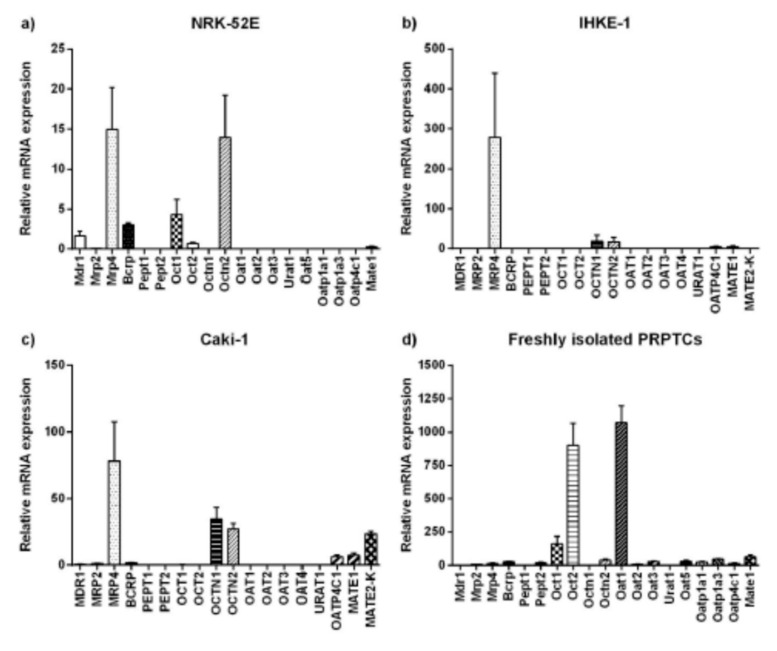
Relative mRNA expression of transport proteins in the renal cell lines NRK-52E (**a**), IHKE-1 (**b**) and Caki-1 (**c**), and in freshly isolated PRPTCs (**d**). The mRNA expression of each gene was normalized to GAPDH/Gapdh expression. Each bar represents the mean ± SD (*n* = 3).

As demonstrated in Figure 9, the expression of transporters varied significantly between the renal cell lines. In NRK-52E cells, the expression of all transporters was comparatively low. The highest relative expression was found for Mrp4 and Octn2. Furthermore, low amounts of mRNA of Mdr1, Bcrp and the organic cation transporters Oct1 and Oct2, as well as Mate1, were detected (Figure 9a). While IHKE-1 cells showed high expression levels of MRP4, mRNA of the other ABC transporters was absent or only minimal amounts could be detected. Moderate expression levels were also found for the SLC transporters OCTN1, OCTN2 and MATE1, as well as for the organic anion transporter OATP4C1 (Figure 9b). Similar to NRK-52E and IHKE-1 cells, the highest relative expression in Caki-1 cells was found for MRP4. In contrast to the other human cell line, IHKE-1, low levels of the other ABC transporters, MDR1, MRP2 and BCRP, could also be detected. With the exception of OCT2, mRNA of all organic cation transporters was found, with OCTN1 and OCTN2 having the highest expression level among them. Moderate levels of MATE1 and MATE2-K could also be detected. The only organic anion transporter found in this cell line was OATP4C1 (Figure 9c). Generally, the renal cell lines NRK-52E, IHKE-1 and Caki-1 did not express the whole set of transporters found in vivo. In particular, organic anion transporters of the SLC22 and SLCO families were almost completely absent. The only exception was OATP4C1, which was found in moderate amounts in the human cell lines IHKE-1 and Caki-1. In marked contrast to this, mRNA of all investigated transporters except for Pept1 was detected in freshly isolated PRPTCs (Figure 9d). The highest expression levels were found for the typical renal organic anion and cation transporters Oat1 and Oct2. Moreover, the freshly isolated PRPTCs expressed several other organic anion transporters, which could not be detected in the cell lines, such as Oat2, Oat3, Oat5, Oatp1a1 and Oatp1a3.

**Figure 10:**
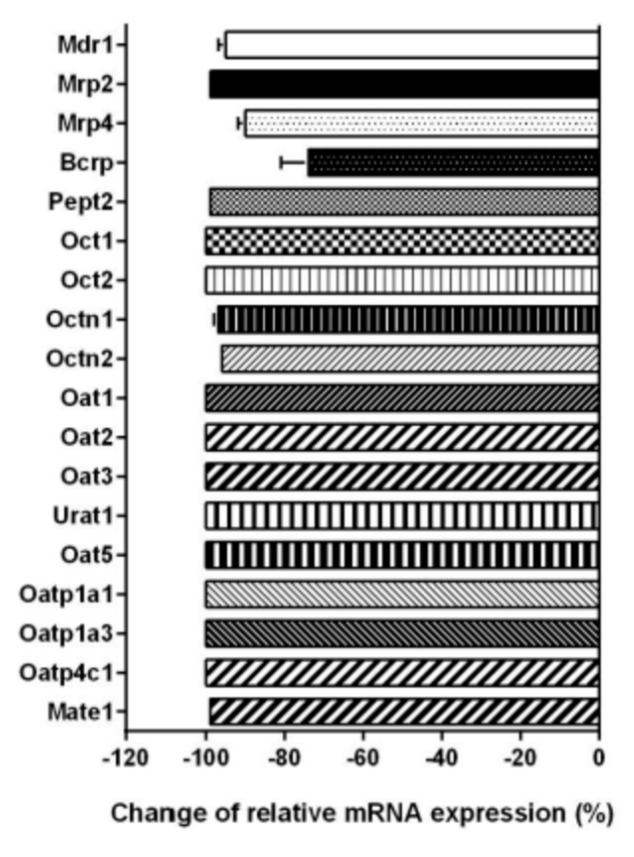
Change in relative mRNA expression of transport proteins in PRPTCs after 5 days in culture. The mRNA expression of each gene was normalized to Gapdh expression and is shown in reference to the expression level of each gene in freshly isolated PRPTCs, which was set to 1. Each bar represents the mean ± SD (*n* = 3).

**Figure 11:**
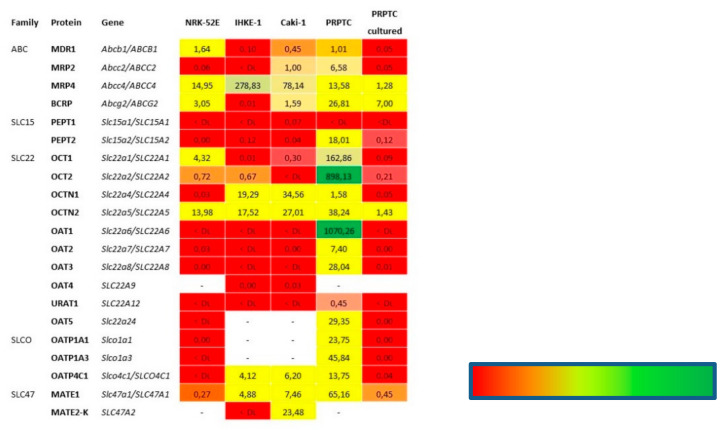
mRNA expression levels of transporters in cell lines normalized to GAPDH/Gapdh expression (*n* = 3). < DL = below detection level, red= low expression intensity, green = high expression intensity.

The relative expression levels of the 18 transporters were also determined in PRPTCs after 5 days in culture. Compared to the freshly isolated PRPTCs, the expression pattern differed notably. Figure 10 shows that the mRNA amount of all transporters was considerably lower after culturing the cells. The lowest reduction was observed as 74% in the case of Bcrp, while the mRNA expression of all other transporters was downregulated by 90–100% compared to the freshly isolated cells. Figure 11 summarizes all transporter expression data.

## 3. Discussion

In vitro cell culture models (e.g., continuous and primary renal cell lines, polarized cell monolayers) of the kidney are attracting increasing interest in the replacement of animal studies. However, the human proximal tubule is a complex, metabolically active epithelial system, and the availability of in vitro cell culture model systems of the human proximal tubular epithelia is very limited. Although they are mostly reductive and lack the complexity of the intact organ, immortalized cell lines and primary cultures of isolated defined segments of the nephron may provide valuable systems for investigating the regulation of a variety of glomerular and renal tubule epithelial cell functions. In this present analysis, we performed a comparative investigation on freshly isolated primary rat proximal tubule cells, primary rat proximal tubule cells cultured for 5 days and the immortalized cell lines MDCKII, NRK-52E, IHKE-1 and Caki-1. From all studies, striking differences between primary cells, cultured primary cells and cell lines became obvious, starting with cell morphology at cell culture (Figure 2), the expression of tight junction proteins (Figure 5, Figure 6 and Figure 7) or completely different patterns of expression of transport proteins (Figure 9). Distinct cell types are certainly suitable to answer specific questions. For example, MCDKII cells, originally derived from the kidney of a cocker spaniel in 1958, belong to the best characterized cell lines with respect to transport studies. They have often been used to overexpress proteins such as p-glycoprotein or breast cancer resistance protein to specifically study excretory transport. However, our own previous studies and those of others [14,15,16,17] demonstrated the rather diverse expression of transport proteins dependent on the subtype of MDCK cells. NRK-52 cells have also widely been used mainly for toxicological studies [18,19,20]; however, to date, detailed cell characteristics have not been published. The same is the case for IHKE-1 cells. Caki-1 cells are well characterized to be of proximal tubule nature due to the presence of γ-glutamyl transferase and E-cadherin, specific proximal tubule and epithelial markers, respectively. In addition, they have been shown to be the first human proximal tubule cell line to express OCTN2 and OCT3 [21,22]. For another human cell line, HK-2, it was reported that it is only of very limited suitability for the in vivo situation; although it expresses MDR1 and at least one MRP transporter, it lacks members of the SLC22 family, such as OAT1, OAT3 or OCT2 [23]. Therefore, this cell line was not investigated in the present study.

Looking at the cell morphology, all investigated cells developed confluent monolayers with a typical cuboidal cell morphology, except for the Caki-1 cell line, which developed a fibroblast-like morphology without confluent monolayers. A similar observation was made by [24]. 

The expression analysis revealed that freshly isolated kidney cells expressed all investigated proximal tubule markers. The distal glycolysis marker Hexokinase-1 was not detected. The renal cell lines were clearly different. In particular, the NRK-52E cells were in a state of dedifferentiation compared to freshly isolated cells. The two human cell lines, IHKE-1 and Caki-1, showed a similar expression pattern. In addition, Hexokinase-1 was expressed, indicating a more distal tubular cell type. The expression and activity of alanine-aminopeptidase-mRNA and γ-glutamyltransferase-1, respectively, were at least 10-times lower than in freshly isolated cells. 

The expression pattern of primary cells changed significantly within 5 days of culture. The expression of all investigated markers decreased by around 96–99%. The simultaneous decrease in γ-glutamyltransferase and the increase in hexokinase can be interpreted as a change from gluconeogenetic (proximal) to glycolytic (distal) activity. However, this change might also be a result of adaptation to the culture conditions [25].

An important quality element of tubular cell cultures is the barrier formation of tight monolayers, which is dependent on the expression and function of claudins and other tight junction proteins. The different cell lines showed remarkable differences, and in the primary cells, the expression of all marker proteins decreased significantly with time. Obviously, the primary cells started to dedifferentiate during culture. However, in accordance with the observed mRNA expression pattern, the only claudin detected in cultured cells by immunofluorescence was claudin-7. Thereby, a clear co-localization with ZO-1 and Occludin was seen, being indicative of the formation of tight junction structures. Indeed, the highest TEER values could be measured for the cultured primary cells. In addition, the paracellular permeation of mannitol across layers of primary cells was lower compared to the permeation across NRK-52E, IHKE-1 and Caci-1 cell layers. 

A look at the expression profile of the investigated transport proteins in freshly isolated primary cells reveals a good correlation with previously published data [26]. However, the expression levels decreased significantly with the culture time, which was paralleled by a decrease in alkaline phosphatase, alanine-aminopeptidase, Villin-1 and γ-glutamyl transferase, all being typical for proximal tubule cells. The kidney cells lines exhibited a quite different expression pattern of the transport proteins. Noticeable was the lack of the organic anion transporters of the SLC22 and SLCO families, which is in accordance with observations by Las et al. [27]. In summary, the expression profiles of the transport proteins in the immortalized cell lines NRK-52E, IHKE-1 and CaKi-1 differed significantly from those in freshly isolated tubule cells. 

Taken together, the results of this study reveal a striking heterogeneity between the investigated cell types with respect to marker enzymes, tight junction proteins and transporters. Obviously, a single cell line cannot be used to predict the in vivo situation and we, therefore, suggest the use of more than one cell line in parallel to address a certain transporter-related scientific question. The use of other in vitro technologies may also be considered, e.g., a hanging drop technology, as suggested by [28], when a type of microorganism is formed in vitro. However, since the focus of our work is on transepithelial transport across a confluent monolayer, the transwell technology, being routinely used in the pharma industry, may be advantageous. Independent of the model that will be used, it has to be taken into account that cell lines might significantly differ from the originating primary cell type, which may be of particular importance when scientific questions with pharmacological questions are addressed. In this present study, the focus was towards the expression of the investigated proteins. Further experiments have now been started to investigate the functional properties of the transporters and to reveal the impact of hormones and growth factors on their expression and function. 

## 4. Materials and Methods

### 4.1. Cell Lines

The following renal cell lines were used: MDCKII (Madin-Darby canine kidney II; Netherlands Cancer Institute, Amsterdam, Netherlands), NRK-52E (Normal rat kidney-52E; Deutsche Sammlung von Mikroorganismen und Zellkulturen, Braunschweig, Germany), IHKE-1 (Immortalized human kidney epithelial-1; Bayer Pharma AG, Wuppertal, Germany) and Caki-1 (Carcinoma kidney-1; American Type Culture Collection, Manassas, VA, USA). MDCKII and NRK-52E cells were cultured in DMEM supplemented with 10% fetal bovine serum, 2 mM glutamine, 100 U/mL penicillin and 100 μg/mL streptomycin. IHKE-1 cells were cultured in DMEM/F-12 (1:1) supplemented with 1% fetal bovine serum, 4 mM glutamine, 1% insulin–transferrin–selenium (Life Technologies, Darmstadt, Germany), 1 mM sodium pyruvate, 15 mM sodium bicarbonate, 15 mM HEPES 36 µg/L hydrocortisone 10 µg/L epidermal growth factor, 100 U/mL penicillin and 100 μg/mL streptomycin. Caki-1 cells were cultured in McCoy’s 5A medium supplemented with 10% fetal bovine serum, 100 U/mL penicillin and 100 μg/mL streptomycin. All cells were cultured at 37 °C in an atmosphere of 5% CO_2_ and 95% relative humidity.

### 4.2. Animals

Male Wistar rats (RccHan:WIST, Harlan Laboratories, Rossdorf, Germany) with a mean body weight of 270 ± 35 g were used. Animals were kept under controlled conditions at constant room temperature of 22 °C and a light/dark cycle of 12 h. They had free access to standard chow (V1534-000 R/M-H, ssniff Spezialitaeten, Soest, Germany) and tap water. All animal experiments were performed according to the guidelines for animal protection of the local authorities. 

### 4.3. Isolation of Proximal Tubular Cells from Rats

In order to isolate primary proximal tubular cells from rats (PRPTCs) a slightly modified collagenase perfusion as described was used [15]. Briefly, kidneys were in situ perfused with 150 mL EGTA solution (500 µM) followed by perfusion with 30 mL Hank’s–HEPES buffer (137 mM NaCl, 5 mM KCl, 0.8 mM MgSO_4_, 0.33 mM Na_2_HPO_4_, 0.44 mM KH_2_PO_4_, 26 mM NaHCO_3_, 25 mM HEPES, pH 7.4) in order to remove Ca^2+^ ions. Subsequently, the kidneys were removed and perfused in a recirculating system for 20 min with collagenase solution (4 mM CaCl_2_, 1.3 mg/mL collagenase type 2 in Hank’s–HEPES buffer). The cortex was mechanically separated and homogenized and the resulting suspension was filtered through cell strainers with 100 µm and 70 µM mesh size. Following this, the suspension was centrifuged for 3 min at 200× *g* and 4 °C and the pellet was resuspended in PRPTC culture medium (DMEM/F-12 (1:1) supplemented with 10% fetal bovine serum, 2 mM glutamine, 1% insulin–transferrin–selenium (Life Technologies, Darmstadt, Germany), 100 U/mL penicillin and 100 μg/mL streptomycin). For experiments with cultivated PRPTCs, the cells were standardly seeded on rat tail type 1 collagen-coated cell culture vessels at a density of 4.0 × 10^4^ cell/cm^2^ and cultured at 37 °C in an atmosphere of 5% CO_2_ and 95% relative humidity for 5 days. 

### 4.4. RNA Expression Analyses

Renal cell lines and PRPTCs were seeded on cell culture flasks and cultured for 5 days under standard conditions. Initially, the cells were removed from the culture vessels by rinsing of the monolayers with PBS and incubation with trypsin–EDTA solution (0.25%) for 5 min. Isolation of total RNA was done with QIAshredder^™^ columns and the RNeasy^®^ Mini Kit (Qiagen, Hilden, Germany) according to the manufacturer’s protocols. RNA quantity and quality were determined by measuring absorbance at 260 nm and 280 nm with a spectrophotometer (NanoDrop^™^ 2000c, peqlab, Erlangen, Germany). Additionally, RNA integrity was assessed by checking 28S and 18S rRNA bands on a denaturing agarose gel stained with ethidium bromide. Isolated RNA was then reverse-transcribed using the QuantiTect^®^ Reverse Transcription Kit (Qiagen, Hilden, Germany) according to the manufacturer’s instructions. For quantification, quantitative real-time PCR (qPCR) was performed using the QuantiTect^®^ SYBR^®^ Green Kit (Qiagen, Hilden, Germany) and a Step One Plus^™^ Real-Time PCR Cycler (Applied Biosystems, Foster City, USA). The utilized primers are shown in Table 3 and Table 4. The PCR condition was 95 °C for 15 min for initial activation, followed by 40 cycles of 94 °C for 15 s, 55 °C for 30 s and 72 °C for 30 s, and a final cycle of 95 °C for 15 s, 60 °C for 60 s and 96 °C for 15 s for melting curve analysis. Relative mRNA expression of each gene was determined by normalization to the expression level of the reference gene glyceraldehyde 3-phosphate dehydrogenase (GAPDH).

### 4.5. Immunofluorescent Staining

Renal cell lines and PRPTCs were seeded on 8-well chambered glass slides (Lab-Tek^®^ Chamber Slides^™^, Thermo Fisher Scientific, Ulm, Germany) and cultured for 5 days under standard conditions. On the day of the experiment, cell monolayers were washed three times with PBS before incubation with methanol at −20 °C for 10 min for fixation and permeabilization. After rinsing again with PBS, the monolayers were incubated with blocking buffer (150 mM NaCl, 16 mM Tris-HCl, 84 mM Tris-base, 0.1% Triton-X, 3% donkey serum) for 90 min to reduce non-specific binding. The blocking buffer was removed and the monolayers were incubated for 90 min at 4 °C with the following primary antibodies: TJP1: rabbit anti-TJP1 (Z-R1), occludin: rabbit anti-OCLN (Z-T22), claudin-2: mouse anti-CLDN2 (12H12), claudin-7: rabbit anti-CLDN7 (ZMD.241) and claudin-10: mouse anti-CLDN10 (4B8A12), all from Life Technologies (Darmstadt, Germany). Subsequently, the monolayers were briefly rinsed with PBS and incubated for another 90 min at 4 °C with Cyanin 3 (Cy3)-conjugated secondary antibodies (anti-rabbit-IgG-Cy3 for the visualization of TJP1, occludin and claudin-7, and anti-mouse-IgG-Cy3 for the visualization of claudin-2 and claudin-10, both from Jackson Immuno Research, Suffolk, UK). After rinsing 3 times with PBS, nuclei were counterstained by incubation with 4′,6-diamidino-2-phenylindole (DAPI) for 5 min at room temperature, and monolayers were rinsed in deionized H_2_O and mounted on the slides with Aqua-Poly/Mount (Polysciences, Eppelheim, Germany). Negative control samples that were incubated without primary antibodies were included in order to check for background labeling. After completion of the immunofluorescent staining, samples were viewed with an Axiovert 200 fluorescence microscope and a LD Plan-NEOFLUAR 20×/0.4 lens. Digital images were taken with an AxioCam MRm CCD camera and processed using the AxioVision software (all from Carl Zeiss, Jena, Germany). All images were taken with an exposure time of 1000 ms for the Cy3 signal and 65 ms for the DAPI signal.

### 4.6. Transepithelial Electrical Resistance and Bidirectional Transport Studies with [^14^C]-Mannitol

Renal cell lines and PRPTCs were seeded on 12-well Transwell^®^ inserts (polyester membrane, 0.4 µM pore size, Corning, Wiesbaden, Germany) at a density of 0.15 × 10^6^ cell/cm^2^ and cultured for 5 days under standard conditions. Transepithelial electrical resistance (TEER) was measured for each well with a volt-ohm meter (Millicell^®^-ERS, Millipore, Billerica, MA, USA) at 37 °C. For the determination of the transcellular flux of [^14^C]-mannitol, bidirectional transport studies from both apical-to-basal (a–b) and basal-to-apical (b–a) directions were conducted at 37 °C. Initially, monolayers were briefly rinsed and equilibrated with transport buffer (128 mM NaCl, 5.4 mM KCl, 1 mM MgSO_4_, 1.8 mM CaCl_2_, 1.2 mM Na_2_HPO_4_, 0.4 mM NaH_2_PO_4_, 4.2 mM NaHCO_3_, 15 mM HEPES, 20 mM glucose, pH 7.2) for 10 min. Next, transport buffer containing 600 µM [^14^C]-mannitol (12.5 KBq/mL) was added to the donor compartment (0.6 mL in the apical (a) or 1.6 mL in the basal (b) compartment for a–b or b–a experiments, respectively). After pre-incubation for 10 min, fresh transport buffer was added to the acceptor compartment to start the assay. Samples were taken from the donor compartment after 0 min and 120 min and from the acceptor compartment after 120 min. All samples were transferred to scintillation vials containing 3.5 mL scintillation cocktail (Biofluor Plus, PerkinElmer, Rodgau, Germany) and radioactivity was measured with a liquid scintillation counter (Tri-Carb^®^ 2900TR, PerkinElmer, Waltham, MA, USA).

The apparent permeability coefficient (P_app_) was calculated by the following equation:

$$P_{app}=\frac{V_{acc}}{A\times c_{don,t0}}\times \frac{∆C_{acc}}{∆t}$$

where *V_acc_* is the buffer volume in the acceptor compartment (mL), *A* is the membrane area (cm^2^), *C_don,t_*_0_ is the substrate concentration in the donor compartment at t = 0 (dpm/mL), and Δ*C_acc_/*Δ*t* is the change in the substrate concentration over time in the acceptor compartment (dpm/mL/s).

The efflux ratio was calculated by the following equation:

$$Efflux\;ratio=\frac{P_{app,b-a}}{P_{app,a-b}}$$

where *P_app,b-a_* is the apparent permeability coefficient from basal to apical (nm/s) and *P_app,a-b_* is the apparent permeability coefficient from apical to basal (nm/s).

### 4.7. Statistics

Data are presented as mean ± SD. Comparisons between two groups were performed with the use of the two-tailed unpaired Student’s *t* test. Differences were considered statistically significant when the calculated *p* value was < 0.05.

## Figures and Tables

**Table 1:** List of cell lines used.

	Name	Species	Origin	Type
MDCKII	Madin-Darby canine kidney II	dog	Kidney epithelium	immortalized
NRK-52E	Normal rat kidney-52E	rat	Kidney epithelium	immortalized
IHKE-1	Immortalized human kidney epithelial-1	human	Fetal kidney cortex	immortalized
Caki-1	Carcinoma kidney	human	Kidney carcinoma	immortalized
PRPTC	Primary rat proximal tubule cells	rat	Proximal tubule	primary

**Table 2:** Summary of expression levels of the investigated tight junction proteins in the renal cell lines MDCKII, NRK-52E, IHKE-1 and Caki-1, and in PRPTCs. Rating of the immunostainings was as follows: +, distinct signal at the cell–cell borders; (+), diffuse signal or signal predominantly in cytoplasm; −, no signal detected.

Protein	Cell Lines				Primary Cells
	MDCKII	NRK-52E	IHKE-1	Caki-1	Cultured PRPTCs
TJP1	+	+	+	(+)	+
Occludin	+	+	+	(+)	+
Claudin-2	+	−	(+)	(+)	−
Claudin-7	+	−	−	−	+
Claudin-10	−	−	−	−	−

**Table 3:** List of primers used for qPCR (Rattus norvegicus).

Gene	Accession Number (RefSeq)	For/Rev	Sequence (5′-3′)	Amplicon Length (bp)
Abcb1a	NM_133401.1	for	CATCTTGGCGGACCTTACGGAGG	183
		rev	ACCTGCATAGCGAAACATTGTGAGC	
Abcc2	NM_012833.1	for	GCGCCCTGGGTGACTGACAAG	166
		rev	CAGTCCTGCCCACTACGCCG	
Abcc4	NM_133411.1	for	ACAGCTTACGGCTACGCGGC	181
		rev	GGCCCGTGGTTGTCTTCCCC	
Abcg2	NM_181381.2	for	AGGCGGAGGCAAGTCTTCGTTG	103
		rev	ATTGGCAGGTTGAGGTGCCC	
Alpl	NM_013059.1	for	GTGGAAGGAGGCAGGATTGACCAC	116
		rev	TTCTGGGAAGTCATGGTGCCCG	
Anpep	NM_031012.1	for	TCTGCCGCTGAGCAACACCC	135
		rev	AGATAGCGCTTCATGGGGCCG	
Cldn10	NM_001106058.1	for	TGTTCCATGACGGGCTGTTCCC	96
		rev	GGCAGCCCCCAATTCATACTTTTGC	
Cldn11	NM_053457.2	for	TTCTCCCCTGTATCCGAATG	157
		rev	AAGCTCACGATGGTGATCTC	
Cldn2	NM_001106846.2	for	AGAACAGCTCCGTTTTCTAGATGCC	160
		rev	TGTTCGCTTGTCTTTTGGCTGCG	
Cldn7	NM_031702.1	for	TGGCCATGTTTGTCGCCACGATG	126
		rev	CCAAAGCAGCAAGACCTGCCACG	
Gapdh	NM_017008.3	for	ATCTTCTTGTGCAGTGCCAGCCTCG	115
		rev	ACTTTGTCACAAGAGAAGGCAGCCC	
Ggt1	NM_053840.2	for	TGAGCTGGCACACCAACGGC	169
		rev	CCGGCAGAACACCTCGCACA	
Hk1	NM_012734.1	for	ACACCTGCTTTTCCGCATCCCC	124
		rev	AGCCGCATGGCGTACAGATAC	
Ocln	NM_031329.2	for	CGCCTCTGGTACCTGAAGTAGCCC	159
		rev	GGTCTGGGTCTGTCCTCTTCGCC	
Slc15a1	NM_001079838.1	for	TCTCTGCCTCTCTCTCACTTGCC	166
		rev	TGTGACAGAGAAGACCACCTCGCC	
Slc15a2	NM_031672.1	for	AAGAGGAACATGCAGAGGCACGG	81
		rev	ACGTGGAAATCAAGCTCCCCGC	
Slc22a1	NM_012697.1	for	GCTGATGGAAGTTTGGCAAGCCC	158
		rev	TGGCCTTTGATTTCCTCCTCCCC	
Slc22a12	NM_001034943.1	for	AGGAACCGAACGGGAACCAAGC	107
		rev	GCCAAAGGCAAACCAGCACAGC	
Slc22a2	NM_031584.1	for	ACTGGTTGTATTTGCTGTGGTTGGC	120
		rev	CTTGGCCTCTGCATATTCTCGGC	
Slc22a24	NM_173302.1	for	TACATGGGCCGCAGAACAACCC	99
		rev	ACGCGGGATCTGCATTTCTTGAGTC	
Slc22a4	NM_022270.1	for	AGGCGGGAAGCATGAGGGACTATG	195
		rev	ACGCGCTGCTCAAGTTCACG	
Slc22a5	NM_019269.1	for	TCCTGTGGCTGACCATATCAGTGGG	132
		rev	ACAGCCAGGCCAACACATAGGC	
Slc22a6	NM_017224.2	for	CCACAGTGATTCGGCAGACAGGC	148
		rev	GCACTGGCGACCACAGGGAC	
Slc22a7	NM_053537.2	for	GGCTGTTGCTGCCCAAAGTTGC	135
		rev	CCTCCTGGGTACTCTTCCTCTCCAC	
Slc22a8	NM_031332.1	for	TTCCCAATGACACCCAGAGGGC	101
		rev	GCTGCACACCAAGTCCCACTCTATC	
Slc47a1	NM_001014118.2	for	CAGCTTCCTGAGTGGTATCCTTGGC	139
		rev	TGCACCCAACGCATTTCCCACC	
Slco1a1	NM_017111.1	for	GATGTGTGATTATGGGCCTGGGG	157
		rev	TGCGTTGGCTTTAAGGTCTGTGTTC	
Slco1a3	NM_030837.1	for	CACTACCTCATTTCCTCATGGGCCG	139
		rev	ACACACTCTGCTGGGTCTTGCG	
Slco4c1	NM_001002024.1	for	CCACGGGATTTGCCACGTTTTTACC	89
		rev	AACAGCCCCTCCAAGTGTTGCC	
Tjp1	NM_001106266.1	for	TTCAGTTCGCTCCCATGACAGGC	132
		rev	TCCTCTCTGCTCCGGAGACTGCC	
Vil1	NM_001108224.2	for	TGCCCAATCGGACCTCAGGC	161
		rev	GCGCCACACCTGCACTTCCC	

**Table 4:** List of primers used for qPCR (Homo sapiens).

Gene	Accession Number (RefSeq)	for/rev	Sequence (5′-3′)	Amplicon Length (bp)
ABCB1	NM_000927.4	for	CTCTTTGCCACAGGAAGCCTGAGC	175
		rev	CTTCAAGATCCATTCCGACCTCGC	
ABCC2	NM_000392.3	for	AACGCACATGAAGAGAGAGCTGC	151
		rev	TTCAACATCTTCCAGGACAAGGGC	
ABCC4	NM_001105515.1	for	ACTTCAGAGCTGGTGCTCACTGG	111
		rev	TGTTTGCCCAGTATGAAAGCCACC	
ABCG2	NM_001257386.1	for	CGATATGGATTTACGGCTTTGCAGC	191
		rev	ACAATCATACAAGCCAAGGCCACG	
ALPL	NM_000478.4	for	CAGGGATAAAGCAGGTCTTGGG	102
		rev	CTTTCTCTTTCTCTGGCACTAAGG	
ANPEP	NM_001150.2	for	ACAGCCAGTATGAGATGGACAGC	104
		rev	CACCACCTTTCTGACATTGCCC	
CLDN10	NM_182848.3	for	TATTCATACTGTCAGGGCTGTGC	135
		rev	CTGCCCATCCAATAAACAGAGCG	
CLDN11	NM_005602.5	for	CTGACTGTTCTTCCCTGCATCC	101
		rev	CAGAGAGCCAGCAGAATGAGC	
CLDN2	NM_001171092.1	for	TGGGAAGGCTAGGGAGTCAAAGGG	197
		rev	TAGAAGACCTGAATGGCCTGTGAGC	
CLDN7	NM_001185022.1	for	GGACAGTGGGTCGCCGAGAGC	182
		rev	AAGGGTTTGGGCCAGGTGAATGC	
GAPDH	NM_001256799	unknown ^a^		95
	NM_002046	unknown ^a^		95
GGT1	NM_001032364.2	for	CCCAGAAGTGAGAGCAGTTGG	95
		rev	CTGCACCCGCTATGATGTCC	
HK1	NM_000188.2	for	CAGATCGAGAGTGACCGATTAGC	170
		rev	ATCTTATCCACAACCGCAGCC	
OCLN	NM_001205254.1	for	AGTTGCGGCGAGCGGATTGG	113
		rev	AGGTGGACTTTCAAGAGGCCTGG	
SLC15A1	NM_005073.3	for	ATGAAGTCGGTGCTTCAGGCAGG	111
		rev	AATGTACTCGGCCCACTGTTTGC	
SLC15A2	NM_001145998.1	for	AGCCATCTCCGACAATCTGTGGC	186
		rev	AACAGAGGCTGCTGAAGGCATGG	
SLC22A1	NM_003057.2	for	CCACATTCGTCAGGAACCTCGG	170
		rev	TGGAAGAAGTAGCGTCACTCCCG	
SLC22A12	NM_144585.2	for	ATCTCCACGTTGTGCTGGTTCGC	129
		rev	CATCTTGGCTGGGATGTCCACG	
SLC22A2	NM_003058.3	for	TAGCAGACAGGTTTGGCCGTAAGC	144
		rev	AGCCTGCTTTGCTGACCAGTCC	
SLC22A4	NM_003059.2	for	TCCATTGGTCTGGTCATGCTGGG	188
		rev	AGCATTCTGTTGTAAGCACCGAGG	
SLC22A5	NM_003060.3	for	GCCAATGGGATTGTTGTGCCTTCC	165
		rev	GCCCACTGATATGGTCATCCACAGC	
SLC22A6	NM_004790.4	for	CATCTACGGTGCTGTTCCTGTGGC	158
		rev	GCGTCTGTTTCCCTTTCCTGGG	
SLC22A7	NM_006672.3	for	TAGCTGTCACCCTGCCTTGTGC	181
		rev	ACTTTGCTCACAGCCTCCTGGC	
SLC22A8	NM_001184732.1	for	AGAAAGGCTCAGCTTGGAGGAGC	128
		rev	GGGAAAGACAGAAGGTCATGCGGC	
SLC22A9	NM_080866.2	for	CCGAGTGGGCAACACACAGATTCC	183
		rev	CAGACTCTAGCAGCCAACTTGAGG	
SLC47A1	NM_018242.2	for	AGCTTCCTCAGTGGCATCCTCG	138
		rev	AGCACCCAGAGCGTTTCCTACC	
SLC47A2	NM_001099646.1	for	CGGTGGCCTTTGTCAATGTCTGC	190
		rev	ATGTGCTGGGTGTTGAGGAAGAGC	
SLCO4C1	NM_180991.4	for	ACTGTCGCTGTCCGAGTTTGAGG	135
		rev	ACTACAATACCTTGCGTGACGGC	
TJP1	NM_003257.3	for	TGCCATTACACGGTCCTCTGAGC	155
		rev	CTGCTTTCTGTTGAGAGGCTGGC	
VIL1	NM_007127.2	for	CATTGTGGTGAAGCAGGGACACG	154
		rev	TGTGACCTCAGCAGTGATCTGGC	

^a^ QuantiTect^®^ Primer Assay Hs_GAPDH_1_SG (Qiagen, Hilden, Germany) was used.

## Data Availability

Data is contained within the article.

## Abbreviations

ABC	ATP-binding cassette
ALPL	Alkaline phosphatase liver/bone/kidney
ANPEP	Alanine aminopeptidase
BCRP	Breast cancer resistance protein
Caki-1	Carcinoma kidney-1
CLDN	Claudin
Cy3	Cyanin 3
DAPI	4′,6-diamidino-2-phenylindole
GAPDH	Glyceraldehyde 3-phosphate dehydrogenase
GGT1	γ-glutamyl transferase 1
HK1	Hexokinase 1
IHKE-1	Immortalized human kidney epithelial-1
MATE	Multidrug and toxin extrusion
MDCK	Madin-Darby canine kidney
MDR1	Multidrug resistance 1
MRP	Multidrug resistance
NRK-52E	Normal rat kidney-52E
OAT	Organic anion transporter
OATP	Organic anion transporting polypeptide
OCLN	Occludin
OCT	Organic cation transporter
OCTN	Organic cation transporter, novel
Papp	Apparent permeability coefficient
PEPT	Peptide transporter
PRPTC	Primary rat proximal tubule cell
qPCR	Quantitative real-time PCR
SLC	Solute carrier
SLCO	Solute carrier organic anion
TEER	Transepithelial electrical resistance
TJP1	Tight junction protein 1
URAT	Urate transporter
VIL1	Villin 1
